# The Effect on Mortality of Fluconazole or Echinocandins Treatment in Candidemia in Internal Medicine Wards

**DOI:** 10.1371/journal.pone.0125149

**Published:** 2015-05-04

**Authors:** Francesco G. De Rosa, Silvia Corcione, Claudia Filippini, Stefania Raviolo, Lucina Fossati, Chiara Montrucchio, Chiara Aldieri, Alessia Petrolo, Rossana Cavallo, Giovanni Di Perri

**Affiliations:** 1 Department of Medical Sciences, Infectious Diseases at Amedeo di Savoia Hospital, University of Turin, Turin, Italy; 2 Department of Anesthesia and Critical Care, City of Health and Sciences, Molinette Hospital, University of Turin, Turin, Italy; 3 Department of Public Health and Pediatric Sciences, Laboratory of Microbiology and Virology, City of Health and Sciences, Molinette, University of Turin, Turin, Italy; Instituto de Salud Carlos III, SPAIN

## Abstract

The incidence of candidemia has increased over the past two decades, with an increased number of cases in Internal Medicine and a prevalence ranging from 24% to 57%. This single-center retrospective study was performed to evaluate the epidemiology and the risk factors associated with mortality of candidemia in patients admitted to Internal Medicine wards (IMWs) of the City of Health and Sciences, Molinette Hospital, Turin, from January 2004 to December 2012. For each patient, demographic, clinical and microbiological data were collected. A case of candidemia was defined as a patient with at least one blood culture positive for *Candida* spp. Amongst 670 episodes of candidemia, 274 (41%) episodes occurred in IMWs. The mortality was 39% and was associated at multivariate analysis with sepsis, cirrhosis and neurologic diseases, whilst removal of central venous catheter ≤48h was significantly associated with survival. In the 77 patients treated with early antifungal therapy the mortality was 29% and was not significantly different with caspofungin or fluconazole, whilst in patients with definitive therapy the mortality was significantly lower with echinocandins compared to fluconazole (11.7% Vs. 39%; p=0.0289), a finding confirmed by multivariate analysis. The mortality was significantly associated with sepsis, cirrhosis and neurologic diseases, whilst CVC removal ≤48h was associated with survival. In patients with early therapy, fluconazole or caspofungin were equally effective. However, echinocandins were significantly more effective as definitive treatment, a finding not explained by differences in treatment delays. Further studies are needed to understand the full potential of these different therapeutic strategies in IMWs.

## Introduction

*Candida* is an important cause of bloodstream infection (BSI) and the incidence of candidemia has increased over the past two decades, due to many factors such as an increasing elderly population, novel and more aggressive immunosuppressive drugs, long-term parenteral nutrition and prolonged use of broad-spectrum antibiotics [1–2]. *Candida* spp. infections represent 80% of overall systemic fungal infections and are now the fourth most prevalent causative agent of nosocomial BSI with a crude and attributable mortality rate ranging from 30% to 81% and 5% to 71%, respectively [3].

Over the past 10 years, a shift has been sometimes described with increasing prevalence of non-*albicans* species, especially in intensive care units (ICUs) and hematological wards, with concern for azole resistance [2,4–6]. An increased prevalence of *C*. *glabrata* has been reported with increased use of echinocandins [7]. A reduced antifungal susceptibility in non-*albicans* Candida species and a correlation with fluconazole prophylaxis has been suggested, moreover intrinsic and emerging resistance to azoles represents a major challenge for empirical therapeutic and prophylactic strategies [8].

Although internal medicine wards (IMWs) represent a significant reservoir for patients with candidemia, few investigators have specifically addressed the risk factors for mortality and the epidemiological aspects of candidemia in this population, with an incidence ranging from 24% to 57% [9–10].

This study was performed to evaluate the epidemiology and the risk factors associated with mortality of candidemia in patients admitted to IMWs of the City of Health and Sciences, Molinette Hospital, in Turin, Italy.

## Materials and Methods

All patients hospitalized with positive blood cultures for *Candida* spp. in IMWs of the City of Health and Sciences, Molinette Hospital, Turin, a 1200-bed Academic Hospital with primary and secondary referral were enrolled in this single center retrospective study during the period January 2004–December 2012. Oncohaematological, solid organ transplant, ICU, obstetric-gynecology paediatrics and trauma wards were excluded from the analysis. The study was approved by the Local Ethical Committee (City of Health and Sciences, Molinette Hospital, Turin). The need for informed consent was waived due to the retrospective nature of the study; data were collected according to the Italian laws on privacy. Each patient was assigned a unique code number prior to statistical analysis

For each patient, demographic, clinical and microbiological data were retrospectively collected. A case of candidemia was defined as a patient with at least one blood culture positive, either central or peripheral, for *Candida* spp. Candida species identification was based on colony morphology on chromogenic agar CHROMagar Candida (CHROMagar, Paris, France). MICs were determined by Sensititre YeastOne using CLSI clinical breakpoints *cut-off* values for susceptibility [11].

Definitive antifungal therapy was defined as administration of antifungal agent with *in vitro* activity against *Candida spp*, administered for ≥48h after knowledge of the positive blood cultures. Early antifungal treatment was defined if an appropriate antifungal was administered within 24h from the first blood culture collection. *Escalation* treatment was considered when antifungal treatment was switched to an echinocandin after initial fluconazole.

The mortality was studied at 28 days after the diagnosis of candidemia. Early onset candidemia (EOC) was considered if candidemia was diagnosed ≤10 days from hospital admission [12].

### Statistical analysis

Data are expressed as means and standard deviation (DDS) for continuous variables and with frequencies and percentages for categorical variables. Chi square test was used for categorical variables; Fisher’s exact test was used in case of low frequency of the considered variable. The relationship between predictive variables was evaluated with a logistic regression analysis. All tests were two-tailed and p ≤0.05 was considered significant. Statistics were studied by SAS program.

## Results and Discussion

During the study period there were 670 episodes of candidemia and 274 (41%) episodes occurred in patients admitted to IMWs, with an incidence of 1.8 episodes/1000 hospital admission or 0.16 episodes/1000 days of hospital stay. The median age was 68 years (SD ± 17); 140 patients (51%) were males and 134 were females (49%). The main demographic characteristics are reported in Table 1. A hospitalization during the six months before the onset of candidemia was reported in 58% of cases. The majority of patients had at least one or more comorbidities at hospital admission and the majority of patients had a urinary (77.7%) or central venous catheter (CVC) (71.2%).

**Table 1:** Univariate Analysis of risk factors for mortality.

Variable, N(%)	Overall, (N = 274)	Survivors,(N = 166)	Non-survivors, (N = 108)	P
Age,years(±SD)	68(±17)	69(±15)	73(±14)	0.0229
Male	140(51)	90(54)	53(49)	0.45
Length of hospital stay,days (±SD)	47(±43)	50.9(±49)	41.8(±32)	0.27
Time from hospital admission to diagnosis, days (±SD)	27(±26)	25(±24)	30.5(±29)	0.035
Time from diagnosis to therapy	2(±2.5)	2.5(±2.8)	2.3(±1.7)	0.59
WBC count/mm^3^m (±SD)	9264(±6471)	8097(±5572)	11065(±7314)	≤.0001
Candida isolates				0.34
*C*.*albicans*	169(61.7)	100(60.2)	69(63.9)	
*C*.*glabrata*	28(10.2)	17(10.2)	11(10.2)	
*C*.*parapsilosis*	55(20)	39(23.5)	16(14.8)	
*C*.*kruzei*	1(0.9)	0	1(0.9)	
*C*.*tropicalis*	13(4.7)	6(3.6)	7(6.4)	
*Candida spp*	8(2.9)	4(2.4)	4(3.7)	
Previous hospitalization within 6 months	161(58.7)	97(58.8)	64(59.3)	0.93
Previous parenteral antibiotic therapy ≤6 months	159(58)	99(60)	60(56)	0.52
Neutropenia	15(5.5)	11(6.8)	4(3.9)	0.33
CVC at diagnosis	195(71.1)	120(72.3)	75(69.4)	0.61
CVC removed ≤48h	119(43.4)	94(82.5)	25(34.7)	≤.0001
Non-invasive ventilation	16(5.8)	9(5.5)	7(6.8)	0.65
Total parenteral nutrition	137(50)	82(51.2)	55(56.2)	0.44
Surgical procedures	68(24.8)	45(27.4)	22(22.3)	0.35
No sepsis	141(51.4)	97(58.4)	44(40.7)	0.03^b^
Sepsis^a^	102(37.2)	54(32.5)	48(44.4)	
Immunosuppressive therapy	41(14.9)	22(13.2)	19(17.9)	0.29
Hemodyalisis	12(4.4)	4(2.4)	8(7.48)	0.04
Cardiovascular diseases	181(66)	105(63.3)	76(70.3)	0.22
Diabetes mellitus	71(25.9)	35(21)	36(33.3)	0.02
Kidney diseases	92(33.6)	57(34.2)	35(32.4)	0.74
Cirrhosis	22(8)	6(3.6)	16(14.8)	0.0009
Neurologic diseases	135(49.2)	71(42.7)	64(59.3)	0.007
Urinary catheter	210(76.6)	121(74.6)	89(86.4)	0.02
**Treatment**				
Early appropriate	77(28.1)	54(32.5)	23(21.3)	0.043
Definitive therapy	150(54.7)	88(53)	62(57.1)	0.47
Definitive with Fluconazole	97(35.4)	59(60.8)	38(39)	0.56
Definitive with Echinocandins	17(6.2)	15(88.2)	2(11.7)	0.0289
*Escalation* therapy (Fluco-caspo)	10(3.6)	8(6.11)	2(3.1)	0.81

^a^Sepsis include 32 patients with severe sepsis and 8 with septic shock.

^b^Refers to sepsis Vs. no sepsis.

*C*. *albicans* was the leading cause of infection (61%) followed by *C*. *parapsilosis* (20%) and *C*. *glabrata* (10%). Candidemia was diagnosed after a mean of 24 ± 28 days after hospital admission and there were 68 patients with EOC (24.8%).

Appropriate antifungal therapy was administered in 227 patients (83%), with fluconazole in 145 patients (64.7%) and echinocandins in 25 patients (11.3%). Amongst patients treated, early treatment was given in 77 patients (34%), mostly with fluconazole (63 patients; 81.8%) followed by caspofungin (9 patients; 11.7%). Early caspofungin was given in six, two and one patients with candidemia due to *C*. *albicans*, *C*. *parapsilosis* and *C*. *kruzei*, respectively. Since 2010 caspofungin was more used as early treatment if compared to the years 2004–2010 (6 patients Vs. 3 patients). In patients treated with definitive therapy, 97 (64.6%) were treated with fluconazole and 17 (11.3%) with echinocandins. Fourtyseven (17%) out of 274 patients did not receive any antifungal treatment.

Amongst patients with early treatment, caspofungin was used in 8 out of nine cases with sepsis, severe sepsis or septic shock; in patients with definitive treatment, echinocandins were used in 14 out of 17 patients in sepsis, severe sepsis or septic shock. The mortality in the eight patients with septic shock was 62.5%.

The overall mortality was 39% (108/274). In the 77 patients treated with early antifungal therapy the mortality was 29% (23), whilst it was 39% with definitive treatment. Definitive therapy with echinocandins was significantly associated with survival (p = 0.0289); no significantly different delay for definitive treatment was observed with fluconazole (mean 2± 2.5) and echinocandins (mean 2±2.4). When no antifungal treatment was administered, the mortality was 49%.

Regarding antifungal resistance, all *C*. *albicans* strains were fully susceptible to fluconazole. All other strains were susceptible to echinocandins and amphotericin B.

### Risk factors associated with mortality in patients with candidemia

Univariate analysis is presented in Table 1. Multivariate analysis showed that removal of CVC ≤48h was associated with survival (OR 0.142; IC 95% 0.068–0.297), whilst sepsis, cirrhosis and neurologic diseases were independent risk factors for 28 days mortality (Table 2).

**Table 2:** Multivariate analysis of risk factors for mortality.

Variable	OR	IC 95%	
Removal of CVC ≤48h	0.142	0.068	0.297
Sepsis ^a^	3.204	1.593	6.446
Cirrhosis	7.959	2.532	25.016
Neurologic disease	2.559	1.290	5.074

^a^Sepsis include 32 patients with severe sepsis and 8 with septic shock.

### Risk factors associated with mortality in patients treated

At univariate analysis, mortality was not significantly different with early therapy with caspofungin or fluconazole (33% Vs. 28%; p = ns). In patients with definitive therapy, the mortality was significantly lower with echinocandins than fluconazole (35% *Vs*. 19%, p = 0.0289).

Multivariate analysis showed that removal of CVC ≤48h (OR 0.129; IC 95% 0.061–0.274) and early antifungal treatment (OR 0.462; IC 95% 0.216–0.998) were associated with survival, whilst age, sepsis, cirrhosis and neurologic diseases were independent risk factors for 28 days mortality (Table 3). Definitive therapy with echinocandins was also associated with survival (OR 0.473; 95% CI 0.222–1.000 (Table 3).

**Table 3:** Multivariate subgroup analysis in patients treated.

Variable	OR	IC 95%	
Early treatment	0.462	0.216	0.988
Removal of CVC≤ 48h	0.129	0.061	0.274
Age	1.024	1.002	1.047
Sepsis ^a^	3.629	1.768	7.450
Cirrhosis	7.913	2.471	25.340
Neurologic diseases	2.618	1.311	5.226
Definitive therapy with echinocandins	0.473	0.222	1.000

^a^Sepsis include 32 patients with severe sepsis and 8 with septic shock.

This paper shows for the first time that echinocandins are associated with significantly higher survival as definitive therapy in IMWs. There are a number of issues that may affect the delay of appropriate antifungal therapy in IMWs, including the number of comorbidities, the duration of hospital admission, the severity of disease which at the end may influence the crude mortality rate in patients with candidemia. It has been reported that the treatment delay after the collection of the first positive blood culture is an independent determinant of hospital mortality [9]. In our analysis only 28% of our patients received early antifungal therapy, mostly with fluconazole, highlighting that a delay in treatment for fungal BSI is very frequent in IMWs and is associated with higher risk of mortality, as previously reported in different studies [10,13]. Moreover, 17% of our patients did not receive any antifungal therapy, a finding that deserve to be aggressively corrected [14].

As stated above, our paper highlights new positive considerations for early and definitive treatment with echinocandins or fluconazole. In a timely setting, that we defined as early treatment, within 24 hours by the blood cultures collection, the mortality was as low as 29% and mortality was not significantly different with fluconazole or caspofungin. Furthermore, when we analyzed the risk factors for mortality only in patients treated, we found that early treatment, at multivariate analysis, was associated with significantly lower mortality. Moreover, with a delay not significantly different from fluconazole, definitive treatment with echinocandins was significantly associated with survival at multivariate analysis.

There are data showing better survival with echinocandins [15–16]. In an individual patient-level quantitative review of randomized trials for treatment of invasive candidiasis with 1915 patients from seven trials, echinocandin treatment was significantly associated with lower mortality (OR, 0.65; 95% CI,.45-.94; p = .02) together with early CVC removal [15]. Interestingly, no data on timing of treatment or the amount of patients in IMWs were given but our data confirm those results. In the other paper with a review on nine years with 1392 episodes of candidemia, a trend toward a higher use of echinocandin was observed together with an increase in better outcomes [16].

There is a strong debate regarding the use of fluconazole and echinocandin as first-line treatment of candidemia. Notwithstanding the non-inferiority and the AI recommendation for both antifungals, the IDSA guidelines stated that fluconazole should be reserved to less severe patients, without haemodynamic instability and/or not recently treated with fluconazole, or with *C*. *parapsilosis* infection [17]. In the latest ESCMID guidelines fluconazole had a CI indication for treatment of candidemia in contrast to AI evidence for echinocandins [18]. Our data show that in the early setting fluconazole and caspofungin are equally effective, but for the first time we show the higher effectiveness of echinocandins as a definitive therapy in IMWs, in line with the ESCMID guidelines where fluconazole was downgraded to CI level. [18]. In a recent paper, fluconazole treatment at the time of blood culture positivity in patients with septic shock due to candidemia, mainly including ICU patients, was non significantly inferior to an echinocandin [19].

Regarding *C*. *parapsilosis* Mora-Duarte et al. reported a higher number of episodes of persistent *C*. *parapsilosis* BSI with caspofungin [20], whereas a numerically lower eradication rate was found by Reboli et al with anidulafungin [21]. With this low-degree of evidence, IDSA guidelines suggested fluconazole over the echinocandin-class drugs for treatment of *C*. *parapsilosis* infection [17]. In a recent published paper, by Fernandez-Ruiz no impact of the type of initial antifungal treatment on the risk of clinical failure was reported in *C*. *parapsilosis* BSI between patients treated with echinocandins and those treated with azoles [22]. These results suggest that the presumed impact of the increased MIC values for echinocandins in *C*. *parapsilosis* should be carefully balanced against the advantages of this class of agents over the azoles (such as fungicidal activity, favorable safety profile, and low potential for drug interactions) when choosing the optimal antifungal therapy. Moreover the use of an echinocandin for the first 72 hours of therapy does not negatively influence the outcome of *C*. *parapsilosis* BSI, despite the lower in vitro activity of this class of antifungals compared to azoles [22].

We found an incidence, ranging from 1.1 to 2.7 per 1000 admissions, similar to those reported in a paper by Bassetti et al. [6] but higher than those reported for other centers such as in Canada (0.45 case per 1,000 admissions) [23] and some European Countries (0.20 to 1.09 case per 1,000 admissions), such as Finland (0.026 to 0.03 case per 1,000 admission) [24–28].

The differences in candidemia rates may be explained by age distributions of populations, differences in health care practices, antibiotic usage and epidemiology of species distribution. Our data confirms that in Italy candidemia is very common in IMWs (41%). According to a recent multicenter study in five tertiary care teaching hospitals in Italy and Spain, candidemia in IMWs accounted for 46.9% of all episodes and a single center study also reported that patients admitted in IMWs had the highest mortality rate (54.1%) [3,9].

In accordance with most of the Italian epidemiological data, our paper showed that candidemia due to *C*. *kruzei* is rare in Italy whilst *C*. *parapsilosis* accounts for the large majority of non-albicans isolates. *C*. *parapsilosis* is the second most prevalent species in our report, and this high incidence was also reported in different Canadian, European and Italian hospitals [23–28]. A multicenter study in Italy and Spain described *C*. *albicans* as the leading etiology (58.4%), followed by *C*. *parapsilosis* complex (19.5%), *C*. *tropicalis* (9.3%) and *C*. *glabrata* (8.3%). Our data also show that CVC removal ≤48h was associated with survival [22], whilst the presence of several comorbidities, especially cirrhosis and neurological diseases, was significantly associated with mortality at multivariate analysis.

Our study has some limitations including the retrospective nature, the low number and the heterogeneity of patients, including comorbidities, the timing and type of treatment, the single center design and the similarities with other epidemiological studies. However, it is representative of the epidemiology of candidemia in IMWs, where sound diagnostic and treatment strategies are still lacking and this is the first study to highlight the role of echinocandins as definitive therapy in IMWs.

## Conclusions

In conclusion, with the limitation of the retrospective nature of this study, our report confirm the frequency of candidemia in IMWs, the high mortality associated and the benefit on survival with early antifungal treatment and CVC removal within 48 hours. Moreover, the mortality was not significantly different with fluconazole and caspofungin in the early setting, but our data show for the first time that echinocandins were significantly more effective than fluconazole as definitive therapy, a finding not explained by differences in treatment delays. Appropriate tools are needed to correctly identify patients at risk of candidemia and further studies are needed to better identify the full benefits of echinocandins in IMWs patients.
