# Correction: The Effect on Mortality of Fluconazole or Echinocandins Treatment in Candidemia in Internal Medicine Wards

**DOI:** 10.1371/journal.pone.0131264

**Published:** 2015-06-22

**Authors:** The PLOS ONE Staff

## Notice of Republication

This article was republished on June 3, 2015, to correct an error in the title. The correct title is: The Effect on Mortality of Fluconazole or Echinocandins Treatment in Candidemia in Internal Medicine Wards. Please download this article again to view the correct version. The originally published, uncorrected article and the republished, corrected article are provided here for reference.

## Supporting Information

S1 File Originally published, uncorrected article. (PDF)

S2 File Republished, corrected article. (PDF)
